# Large ovarian cyst in a prepubertal girl associated with severe primary hypothyroidism: a case report

**DOI:** 10.1097/RC9.0000000000000617

**Published:** 2026-06-24

**Authors:** Maysam Jehad Yousef Hamarsheh, Saad Mufid Ali Mohammad, Jeries Eid Abusahlia

**Affiliations:** a Obstertics and Gynaecology Residency Program, Ibn Sina Hospital, Jenin – West bank, Palestinian Territory; b Pediatrics Residency Program, Sheikh Khalifa Medical City, Abu Dhabi-UAE

**Keywords:** adnexal mass, hypothyroidism, pediatric ovarian cyst, Van Wyk–Grumbach syndrome

## Abstract

**Introduction and importance:**

Ovarian cysts in prepubertal girls are uncommon and often raise concerns about torsion or malignancy. Severe primary hypothyroidism can cause ovarian enlargement and cyst formation as part of Van Wyk–Grumbach syndrome.

**Case presentation:**

A 7-year-old prepubertal girl presented with acute lower abdominal pain. Imaging revealed a large, mildly complex left ovarian cyst, a smaller contralateral cyst, preserved vascularity, and no torsion. Laboratory evaluation showed profound primary hypothyroidism, elevated prolactin and estradiol levels, and delayed bone age. Due to the symptoms, cyst size, and mildly complex imaging findings, laparoscopic cyst excision was performed, confirming a benign cyst without torsion or malignancy. Following thyroid replacement therapy, endocrine parameters normalized, and the contralateral cyst regressed.

**Clinical discussion:**

Although many ovarian cysts related to Van Wyk–Grumbach syndrome regress with levothyroxine alone, this case highlights the management dilemma when the presentation is acute and imaging raises concerns about complications.

**Conclusion:**

Hypothyroidism should be included in the differential diagnosis of ovarian cysts in prepubertal girls. Routine thyroid function testing may guide early diagnosis, enable conservative management when appropriate, and help avoid unnecessary surgery.

## Introduction

Ovarian cysts in prepubertal girls are uncommon and raise concerns for torsion, malignancy, and acute abdomen. Although most are functional or benign, endocrine causes should be considered, particularly when cysts are bilateral, large, or disproportionate to pubertal status. Severe primary hypothyroidism can cause ovarian enlargement and cyst formation, known as Van Wyk–Grumbach syndrome. Recognition is important because cyst regression after thyroid hormone replacement has been reported, and early diagnosis may prevent unnecessary ovarian surgery.

We report a 7-year-old prepubertal girl with acute abdominal pain and a large, mildly complex ovarian cyst in the setting of profound primary hypothyroidism. The relevance of this case is the acute presentation, cyst size, contralateral cyst, and the management dilemma created by a reversible endocrine etiology in a child evaluated for possible surgical pathology. This case has been reported in line with the SCARE criteria^[^1^]^.

HIGHLIGHTS

- Ovarian cysts are rare in prepubertal girls and may mimic surgical emergencies.
- Severe primary hypothyroidism can cause ovarian cysts as part of Van Wyk–Grumbach syndrome.
- Thyroid function testing is essential in pediatric adnexal masses.
- Large, symptomatic, or mildly complex cysts may require individualized surgical decision-making.
- Early endocrine recognition may prevent unnecessary surgery and support ovarian preservation.

## Case presentation

A 7-year-old prepubertal girl, previously healthy with normal growth and development, presented with 3 days of acute lower abdominal pain. The pain was sudden, predominantly suprapubic with occasional radiation to the left lower quadrant, intermittent, and severe enough to limit daily activities. There was no fever, vomiting, diarrhea, constipation, urinary symptoms, or vaginal bleeding. Her appetite was mildly reduced, with no documented weight loss.

There was no recent infection, trauma, similar previous episodes, medication use, or known drug allergies. Family history was notable for a maternal aunt with recurrent benign mucinous ovarian cysts, with no family history of thyroid disease, malignancy, or endocrine disorders.

On examination, vital signs and anthropometric measurements were appropriate for age. Abdominal examination showed mild suprapubic and left lower quadrant tenderness without guarding, rebound tenderness, or palpable mass. Other systems were unremarkable. Tanner Stage I confirmed prepubertal status.

Urgent imaging was performed to evaluate appendicitis, ovarian torsion, or other surgical causes.

Ultrasonography showed a well-defined cystic lesion with a few internal septations, measuring 7 × 4.6 cm in the lower mid-pelvis, closely related to the uterus and suggestive of an ovarian cystic lesion. Minimal pelvic free fluid was noted, the appendix was normal, and the solid organs were unremarkable.

Contrast-enhanced CT revealed a mildly complex left ovarian cyst measuring 8.5 × 6.2 × 6.8 cm with thin septations, a right ovarian cyst measuring 3 × 2.4 cm, and mild pelvic free fluid. Doppler showed preserved ovarian vascularity with no torsion. No lymphadenopathy, solid enhancing component, papillary projection, or radiological feature strongly suggestive of malignancy was reported.

## Laboratory investigations

Hormonal evaluation revealed:

- Thyroid-stimulating hormone (TSH): >100 mIU/L
- Free thyroxine (free T4): 0.4 ng/dL
- Free triiodothyronine (free T3): <1.07 pg/mL
- Follicle-stimulating hormone (FSH): 8.27 IU/L
- Luteinizing hormone (LH): 0.00 IU/L
- Prolactin: 50 ng/mL
- Estradiol: 36 pg/mL

Bone-age assessment showed a markedly delayed bone age of 3 years at a chronological age of 7 years.

Hematological investigations showed:

- White blood cell count: 6.1 × 10^3^/µL
- Hemoglobin: 10.7 g/dL
- Platelet count: 330 × 10^3^/µL

Overall, profound primary hypothyroidism, elevated prolactin and estradiol, prepubertal status, bilateral ovarian cysts, and delayed bone age are consistent with Van Wyk–Grumbach syndrome.

## Management and outcome

Conservative management with thyroid hormone replacement alone was considered because ovarian cysts associated with Van Wyk–Grumbach syndrome may regress with levothyroxine. However, surgery was selected because the child had acute, activity-limiting pain, a large dominant cyst, mildly complex septated features, and concern for torsion or rupture despite preserved Doppler flow. Malignancy was less likely radiologically but could not be excluded solely by endocrine findings.

The patient underwent laparoscopic exploration. A simple left ovarian cyst measuring approximately 7 cm was identified, with no torsion, necrosis, peritoneal deposits, concerning ascites, or gross malignant features. The cyst was evacuated, followed by complete cyst wall excision. Hemostasis was achieved, and the procedure was uneventful.

The postoperative course was uncomplicated, and the patient was discharged the same day. She was referred to pediatric endocrinology and started on thyroid hormone replacement. At the 6-month follow-up, thyroid function tests had normalized, prolactin and estradiol levels had returned to normal, and pelvic ultrasonography showed regression of the contralateral ovarian cyst.

## Discussion

Ovarian cysts in prepubertal girls are uncommon and may prompt urgent surgical evaluation because the differential diagnosis includes functional cysts, benign neoplasms, malignancy, torsion, and endocrine-related ovarian enlargement^[^2–4^]^.

Appendicitis was less likely after normal appendiceal imaging and the absence of inflammatory features. Torsion was considered because of acute pain and cyst size; however, preserved Doppler flow and the absence of intraoperative torsion argued against it, while recognizing that Doppler flow does not fully exclude intermittent torsion^[^5^]^. Malignancy was less likely given the absence of solid components, papillary projections, lymphadenopathy, abnormal solid-organ findings, or gross malignant features. Bilateral cysts, profound hypothyroidism, and prepubertal status supported hypothyroidism-associated ovarian stimulation.

Van Wyk–Grumbach syndrome is a rare manifestation of long-standing untreated hypothyroidism, characterized by ovarian cysts, ovarian enlargement, isosexual pseudoprecocity, delayed bone age, and occasionally galactorrhea^[^6–8^]^. The mechanism likely involves hormonal overlap, where markedly elevated TSH exerts FSH-like activity through shared alpha subunits, causing ovarian stimulation and cyst formation^[^6,8^]^. Hyperprolactinemia may contribute^[^7,8^]^. Elevated prolactin, estradiol, and delayed bone age strengthen the diagnosis.

Many reported cases show complete or near-complete cyst regression after levothyroxine alone^[^7,9^]^. Similarly, this patient demonstrated biochemical recovery and contralateral cyst regression after thyroid hormone replacement, supporting hypothyroidism as the underlying driver.

Conservative treatment is appropriate for stable patients when torsion, rupture, and malignancy are unlikely, and follow-up can be ensured. In contrast, this patient had acute pain, a cyst larger than 8 cm, mild complexity, and a smaller contralateral cyst. Laparoscopy was, therefore, used to control symptoms, evaluate the adnexa, exclude urgent pathology, and preserve ovarian tissue.

This case emphasizes the value of endocrine evaluation, including thyroid function, prolactin, estradiol, and bone age, in suspected Van Wyk–Grumbach syndrome. These tests help distinguish endocrine-related ovarian enlargement from other pediatric adnexal masses and may support conservative management when clinically safe.

Awareness of hypothyroidism as a reversible cause of ovarian enlargement is essential. Including thyroid function testing in pediatric adnexal mass evaluation may enable earlier diagnosis, avoid unnecessary surgery, and support fertility-preserving management^[^2,3,9^]^.

## Conclusion

Severe primary hypothyroidism should be considered as a reversible cause of ovarian cysts in prepubertal girls. In this patient, profound hypothyroidism, elevated prolactin and estradiol, delayed bone age, bilateral cysts, and contralateral cyst regression after thyroid replacement supported Van Wyk–Grumbach syndrome.

Routine thyroid function testing can facilitate early diagnosis and conservative treatment with levothyroxine when appropriate. However, surgery may still be warranted in cases of significant pain, large cyst size, or indeterminate imaging, emphasizing individualized judgment and follow-up.

Multidisciplinary care is essential to balance timely intervention with ovarian preservation.

## Data Availability

Not applicable.
